# Reproduction Immunity Trade-Off in a Mollusk: Hemocyte Energy Metabolism Underlies Cellular and Molecular Immune Responses

**DOI:** 10.3389/fphys.2019.00077

**Published:** 2019-02-11

**Authors:** Katherina Brokordt, Yohana Defranchi, Ignacio Espósito, Claudia Cárcamo, Paulina Schmitt, Luis Mercado, Erwin de la Fuente-Ortega, Georgina A. Rivera-Ingraham

**Affiliations:** ^1^Laboratory of Marine Physiology and Genetics, Centro de Estudios Avanzados en Zonas Áridas, Universidad Católica del Norte, Coquimbo, Chile; ^2^Centro de Innovación Acuícola AquaPacífico, Universidad Católica del Norte, Coquimbo, Chile; ^3^Magister en Ciencias del Mar, Universidad Católica del Norte, Coquimbo, Chile; ^4^Grupo de Marcadores Immunológicos, Laboratorio de Genética e Immunología Molecular, Instituto de Biología, Pontificia Universidad Católica de Valparaíso, Valparaíso, Chile; ^5^Departamento de Ciencias Biomédicas, Facultad de Medicina, Universidad Católica del Norte, Coquimbo, Chile; ^6^Laboratoire Environnement de Petit Saut, HYDRECO, Kourou, French Guiana

**Keywords:** reproductive cost, scallop immunity, hemocyte metabolism, hemocyte respiration, scallop immune genes

## Abstract

Immune responses, as well as reproduction, are energy-hungry processes, particularly in broadcast spawners such as scallops. Thus, we aimed to explore the potential reproduction-immunity trade-off in *Argopecten purpuratus*, a species with great economic importance for Chile and Peru. Hemocytes, key immunological cells in mollusks, were the center of this study, where we addressed for the first time the relation between reproductive stage, hemocyte metabolic energetics and their capacity to support immune responses at cellular and molecular levels. Hemocyte metabolic capacity was assessed by their respiration rates, mitochondrial membrane potential and citrate synthase (CS) activity. Cellular immune parameters such as the number of circulating and tissue-infiltrating hemocytes and their reactive oxygen species (ROS) production capacity were considered. Molecular immune responses were examined through the transcriptional levels of two pattern recognition receptors (*ApCLec* and *ApTLR*) and two anti-microbial effectors (*ferritin* and *big defensin*). Their expressions were measured in hemocytes from immature, matured and spawned scallops under basal, and one of the following challenges: (i) *in vitro*, where hemocytes were challenged with the β glucan zymosan, to determine the immune potentiality under standardized conditions; or (ii) *in vivo* challenge, using hemocytes from scallops injected with the pathogenic bacteria *Vibrio splendidus*. Results indicate a post-spawning decrease in the structural components of the immune system (hemocyte number/quality) and their potential capacity of performing immune functions (with reduced ATP-producing machinery and exhaustion of energy reserves). Both *in vitro* and *in vivo* challenges demonstrate that hemocytes from immature scallops have, in most cases, the best metabolic potential (increased CS activity) and immune performances, with for example, over threefold higher ROS production and tissue-infiltration capacity than those from mature and spawned scallops after the bacterial challenge. Agreeing with cellular responses, hemocytes from immature individuals induced the highest levels of immune receptors and antimicrobial effectors after the bacterial challenge, while spawned scallops presented the lowest values. Overall, results suggest a trade-off between resource allocation in reproduction and the immune responses in *A. purpuratus*, with hemocyte energy metabolic capacity potentially underlying cellular and molecular immune responses. Further research would be necessary to explore regulatory mechanisms such as signaling pleiotropy which may potentially be underlying this trade-off.

## Introduction

Both reproduction and immune defense are fitness associated traits with high energy demand (Lochmiller and Deerenberg, 2000; Schwenke et al., 2016). Because life-history evolution tends to optimize rather than maximize energy resources, reproduction, and immune response can be mutually constraining (Schwenke et al., 2016). Therefore, a trade-off between these two relevant processes may arise as a consequence of limiting energy availability.

Because scallops are broadcast spawners, they invest massively in reproduction to produce abundant energy-rich gametes in order to ensure fertilization success, and to allow early planktonic development without external food. In this group of bivalves, energy investment in reproduction has shown to reduce other vital processes such as swimming ability and escape response capacity (Brokordt et al., 2000a,b, 2003, 2006; Kraffe et al., 2008), and tolerance to environmental stress (Brokordt et al., 2015). In addition, post-reproduction mass mortalities have been reported for scallops and other marine bivalves (Tremblay et al., 1998; Xiao et al., 2005; Samain et al., 2007). For oysters, it has been proposed that mass mortality is the result of multiple factors including physiological imbalance associated with reproduction, aquaculture practices, and increased susceptibility to pathogens (Samain et al., 2007).

For a given animal, defense against pathogens is determined in part by its immune response capacity. Scallop’s immune defense, like all invertebrates, relies on innate immunity mechanisms because they lack an adaptive immune system. Thus, they have developed a series of sophisticated and effective strategies to recognize and eliminate invading microorganisms (Loker et al., 2004; Song et al., 2015). These strategies include cascades of cellular and humoral immune responses that have been recently shown to be coordinated by a series of extracellular stimuli or messengers, cell receptors, signaling pathways and the regulation of the expression of antimicrobial effectors (Song et al., 2015; Oyanedel et al., 2016a,b).

Hemocytes in particular are involved in various physiological functions including nutrient storage and transport and tissue repair (Beninger et al., 2003; Donaghy et al., 2009), all processes which may be increased during gonad maturation (e.g., nutrient transport) and after spawning (e.g., gonad resorption). However, being the only circulating cells in mollusks, they also play a key immunity role, acting as sentry cells to induce the immune response. Hemocyte defense mechanisms include: (1) chemotaxis, (2) recognition of non-self-particles by soluble and membrane bound receptors, (3) activation of intracellular signaling cascades, (4) opsonization (i.e., secretion of soluble factors toward extracellular environment), (5) phagocytosis, and (6) intracellular degradation of foreign material (Donaghy et al., 2009). The chemotaxis process includes an active migration and infiltration of hemocytes toward the affected tissues. In vertebrates, it has been shown that mounting the initial immune response demands high levels of nutrients and energy due to the hypermetabolic state of the (energy-hungry) phagocytic immune cells (macrophages) involved (Lochmiller and Deerenberg, 2000). Also in invertebrates it was observed that the immune defense is energetically costly, requiring metabolic adaptation and reallocating energy from different tissues toward the immune system (Bajgar et al., 2015). In the scallop *Chlamys farreri* for example, mounting the immune defense against the pathogenic bacterium *Vibrio anguillarum* was observed to be mainly at the expense of glycogen stored in the adductor muscle and the digestive gland (Wang et al., 2012).

For bivalve molluscs, reproduction-immunity trade-offs have been investigated mainly in oysters, though solely through the assessment of cellular immune parameters (Cho and Jeong, 2005; Li et al., 2007; Samain et al., 2007; Wendling and Wegner, 2013). In contrast, this trade-off has been widely addressed in insects (reviewed by Schwenke et al., 2016). The main conclusions of these studies are that (i) physiological costs of reproduction frequently involve the decrease in both basal and induced levels of immunity and (ii) that the energetic requirements of reproduction and immunity indicate that the reallocation of a common energy source may be the basis for the trade-off between these traits (Schwenke et al., 2016). It is however important to remark that none of these studies have evaluated the reproduction-immunity trade-offs considering the various components of the reaction cascade associated with the immune response.

The scallop *Argopecten purpuratus* is one of the most cultured molluscs in countries such as Chile or Peru. In the former, collection of wild *A. purpuratus* is prohibited, and aquaculture production reached 19,018 tons by year 2000. In Peru, the production of this scallop represents the main aquaculture product of the country, and by 2014 represented 45,300 tons, i.e., 56.4% of the total aquaculture production of the country (PromPerú, 2014). However, its production in these countries has gradually declined in part due to the increasing number of mass mortality events. In Chile alone, for the period 2000–2016 this decrease overpassed 84% of the total production (FAO, 2016). As in other bivalves, these mortality events usually coincide with the reproductive period but its causes have not been yet elucidated. Pathogenic infections cannot be ruled out as being partly responsible given that several studies have shown that vibriosis produces massive mortalities in *A. purpuratus* hatchery-reared larvae (Riquelme et al., 1996, 2000; Rojas et al., 2015). While *Vibrio splendidus* infection is still mainly recognized as a larval problem in *A. purpuratus*, it has more recently been identified as a pathogenic agent with fatal consequences for adult *Patinopecten yessoensis* scallops (Liu et al., 2013).

In this study, we aimed to explore a potential reproduction-immunity trade-off in *A. purpuratus*, contributing to the knowledge in (non-oyster) bivalves and in a species with great economic importance for countries such as Chile or Peru. Furthermore, we address for the first time this subject using a multi-approach methodology, avoiding relying solely on cellular immune parameters to have an overview of relation between bio-energetics and immunity/reproduction. To do this, *A. purpuratus* in different reproductive stages (immature, maturing, and spawned) were challenged with *V. splendidus*. Basal immunity and the capacity to mount the immune response after a challenge was assessed at the cellular level and at the transcriptional levels of immune genes encoding immune sensors and antimicrobial effectors. Considering that mollusk hemocytes have an active participation in immune response but also contribute in transporting nutrients to the growing gonad (Beninger et al., 2003), reproduction-immunity trade-offs may not only be energetic but also functional. Therefore, in this study we paid especial attention to the relation between the reproductive status on the hemocyte capacity to function as immune cells, by evaluating their metabolic capacity, and phagocytic [through reactive oxygen species (ROS) production] and tissue infiltration capacities after the challenge. Finally, these results were correlated with metabolic parameters, i.e., hemocyte respiration and mitochondrial membrane potential (Δωm). Overall, the results of this study will contribute to comprehend the potential reasons behind the increasingly frequent mortality events of *A. purpuratus* cultures and design adequate measures contributing to reduce the economic loss entailed by such events.

## Materials and Methods

### Scallop Procurement and Holding Conditions

Adult *A. purpuratus* (70–80 mm shell height) with immature and mature gonads were obtained from the aquaculture facilities of the Universidad Católica del Norte (UCN) located at the Tongoy Bay in Coquimbo. It should be noted that as gonad maturation in *A. purpuratus* is somehow asynchronous, it is possible to simultaneously obtain scallops at different reproductive stages. Scallops were transported to the UCN laboratory in Coquimbo, and acclimated to laboratory conditions for 4 days, in 1,000 L tanks supplied with filtered, aerated, running seawater, and fed a diet composed of *Isochrysis galbana* and *Chaetoceros calcitrans* in equal amounts. Following acclimation, a group of mature scallops were stimulated to spawn by adding excess microalgae.

### Bacteria Procurement

A pathogenic strain of *V. splendidus* (VPAP18) for *A. purpuratus* larvae (Rojas et al., 2015) was kindly donated by Dr. Rojas from the Microbiology Lab at UCN. The strain was maintained in Trypticase Soy Agar NaCl 3% and grown in Trypticase Soy broth NaCl 3% until exponential phase (DO_600_: 0.2–0.4). Bacteria was then washed three times by centrifugation with 0.22 μm filtered sterilized seawater (SSW) and reconstituted in SSW at a DO_600_ of 0.09, corresponding to 1 × 10^7^ CFU (colony forming units) × mL^-1^ as determined empirically in our laboratory. Bacterial concentrations were confirmed by a standard dilution plating technique.

### Experimental Setup

Scallops from each reproductive stage (immature, mature, and spawned; *n* = 24 per reproductive status) were injected in the adductor muscle with either 100 μL of a sublethal dose of the VPAP18 strain (1 × 10^7^ CFU × mL^-1^ as determined by preliminary LD50 assays) (*n* = 8 per reproductive condition), or with 100 μL of 0.22 μm filtered SSW, serving as injury control (*n* = 8 per reproductive condition). In parallel, naïve (undisturbed) adult scallops were also considered for assessing basal levels of immune parameters at each reproductive stage (*n* = 8 scallops per reproductive condition).

Hemolymph from each scallop was extracted from the adductor muscle 24 h post-injection. Preliminary assays showed that after 24 h post-challenge *A. purpuratus* presents the highest induction of immune response. One fraction of the hemolymph was used to determine the number of circulating hemocytes (TCH), their capacity to produce ROS, and their respiration rate capacity. The other hemolymph fraction was used to isolate hemocytes through centrifugation (600 × *g* for 5 min at 4°C). The resulting pellet was split into two parts and immediately frozen in liquid nitrogen and stored at -80°C. These hemocyte pellets were used for the analyses of the mitochondrial enzyme citrate synthase (CS) and protein content and for the analyses of gene transcriptional levels by RT-qPCR, respectively.

For each of the three reproduction stages and for naïve scallops only, we further used a small aliquot of hemolymph to carry out an *ex vivo* challenge of hemocytes as detailed later on (see section “Respiration Rates”). For these challenged hemocytes, we conducted respiration rate (RR) and mitochondrial membrane potential (Δωm) measurements.

### Hemocyte Metabolism

#### Respiration Rates

Hemocyte RR were determined on fresh hemolymph samples collected from the adductor muscle of experimental scallops using a 3 mL cold syringe (needle 23G X 1”). Two types of respiration measurements were carried out using animals under the three different reproductive stages: (i) *ex vivo* challenge, using naïve scallops which hemocytes were challenged with zymosan and (ii) *in vivo* challenge, using hemolymph samples from scallops subjected to the bacterial or SSW injection (as described in section “Experimental Setup”). For the first, samples consisted in 81 μL hemolymph to which 9 μL of either zymosan (zymosan A from *Saccharomyces cerevisiae*, Sigma, prepared in 1X PBS to a final concentration of 16 particles per hemocyte) or 1X PBS (for control purposes) was added. For the second, samples consisted of 90 μL of undiluted hemolymph. The *ex vivo* challenge aimed to assess the potential metabolic capacity to mount the defense response by the hemocytes from scallops at the three reproductive stages. We further reasoned that by exposing the hemocytes to a MAMP (microbe-associated molecular pattern) such as the β glucan zymosan, the tests would better allow comparing responses under controlled standardized conditions. On the other hand, the *in vivo* challenge with the bacteria would allow analyzing the metabolic cost of mounting the immune response under more realistic cellular/physiological conditions; and considering the time at which the associated immune molecules are induced.

To determine hemocyte RR, 90-μL glass metabolic chambers equipped with an oxygen sensor spots (OXSP5, sensor code SD7-541-207, Pyro-Science GmbH, Aachen, Germany) glued to the inner side of the chamber were used. Measurements were carried out as in Rivera-Ingraham et al. (2016). Briefly, chambers were filled with an hemolymph sample and were then closed, ensuring the absence of any air bubbles within the chamber and measurements were carried out at 20°C using a four-channel fiber optic oxygen meter (Firesting, Pyro-Science GmbH). All measurements started at an O_2_ partial pressure (*p*O_2_) of around 70–80%, the natural air saturation of the hemolymph sample after extraction and transfer to the metabolic chamber. The O_2_ concentration in each of the chambers was registered each 5 s through the Pyro Oxygen Logger software as a functioning of declining *p*O_2_. Four measurements were recorded in parallel, where hemocytes were allowed to respire until O_2_ was completely consumed in the chamber, a process which took no more than 2 h. Between four and eight replicates were carried out for each developmental stage and experimental treatment. When possible, the critical *p*O_2_ (*p_c_*O_2_), as defined by Tang (1933) and indicating the onset of anaerobic metabolism, was calculated using the equation by Duggleby (1984). RRs were calculated as a function of declining *p*O_2_. Given the oxyconformity behavior of hemocytes, for statistical purposes RRs between 30 and 40% *p*O_2_ were calculated by linear regression on plots representing oxygen concentration over time in the chamber. The 30–40% *p*O_2_ interval was chosen as representative of the natural *p*O_2_ in bivalve hemolymph according to the reports of Allen and Burnett (2008) and Handa et al. (2017, 2018). For each hemolymph sample tested, the number of hemocytes was quantified using a Neubauer cell-counting chamber to express RRs as nmol O_2_ ⋅ min^-1^ ⋅ million hemocytes^-1^.

#### Citrate Synthase Activity

Apparent specific activity of the key mitochondrial enzyme CS was measured in hemocyte pellets from each scallop 24 h after the injections. The pellets were manually homogenized on ice in 8 volumes of homogenization buffer (pH 7.2) containing 50 mM imidazole-HCl, 2 mM EDTA-Na (ethylene dinitrilotetraacetic acid), 5 mM EGTA (ethyleneglycol 2 tetraacetic acid), 150 mM KCl, 1 mM dithiothreitol and 0.1% Tween-20. The homogenates were then centrifuged at 600 *g* at 4°C for 10 min. Conditions for enzyme assays were adapted from those used by Brokordt et al. (2000a) as follows (all concentrations in mmol L^-1^): TRIS-HCl 75, oxaloacetate 0.3 (omitted for the control), DTNB (5,5-dithio-*bis*-2-nitrobenzoic acid, Ellman’s reagent) 0.1, acetyl CoA 0.2, pH 8.0. Enzyme activities were measured at 20°C using a microplate spectrophotometer EPOCH (BioTek) to follow the absorbance changes at 412 nm to detect the transfer of sulfydryl groups from CoASH to DTNB. The molar extinction coefficients used for DTNB was 13.6. All assays were run in duplicate and the specific activities expressed in international units (IU, μmol of substrate converted to product per min) per mg of hemocyte wet mass.

#### Hemocyte Mitochondrial Membrane Potential

Hemocyte Δωm was determined in fresh hemolymph samples, collected as previously described, from eight undisturbed scallops for each of the three developmental stages considered (*n* = 24). The sample was aliquoted into two inverted-microscope slides (30 μL per slide), which were maintained in humid chambers until their analysis to avoid sample evaporation. It was in these slides and in humid conditions that hemocytes were allowed to adhere on the microscope slides for 10 min, time after which, one of the aliquots was treated with 5.3 μL of zymosan (final concentration of 16 particles per hemocyte in SSW) while the other received the equivalent volume of SSW. After 15 min, the fluorophore JC-10 (52305, Enzo Life Sciences) diluted in DMSO was added to each of the two aliquots to obtain a final concentration of 5 μM (Donaghy et al., 2012). This fluorophore accumulates in mitochondria and exists under monomer form under low Δωm conditions. JC-10 is excitable with an Ar lasser (488 nm), and while monomers exhibit a green fluorescence, JC-10 aggregates (occurring under higher Δωm conditions), exhibit a red fluorescence. Due to hemocyte morphology and the fact that these may aggregate, a criteria for a common confocal plane was established. In the present study we used the plane of focus of hemocyte nuclei, and for this, the nucleus-staining fluorophore Hoechst 33342 (Chazotte, 2011; Invitrogen) was equally added to each sample (2 μL of a stock solution 1:100 in water). Samples were incubated with these two fluorophores simultaneously during 30 min, and were then visualized in a Zeiss LSM 800 confocal microscope (Carl Zeiss, Heidelberg, Germany) equipped with diode lasers. Visualization and imaging was carried out using a Plan-Neofluar 63x/1.3 Imm Korr objective. To avoid photobleaching, the areas of interest (i.e., those containing healthy looking, moderately packed and adhered hemocytes) were located using transmission light. Then, and for each of these selected areas, three single pictures were taken, with the following conditions and in the following order (increasing excitation energy): (i) Ex: 493 nm, Em: 410–533 nm to register the green fluorescence corresponding to JC-10 in monomer form (JC_mon_); (ii) Ex: 488 nm, Em: 550–600 nm to register the red fluorescence corresponding to JC-10 in its aggregate form (JC_agg_), and (iii) Ex: 345 nm, Em: 400–467 nm to visualize hemocyte nuclei. Picture resolution was in all cases 512 × 512 pixels and 16 bit.

For each picture taken, only hemocytes with their nuclei in clear plane of focus (as evidenced by the Hoechst fluorescence) were considered for analysis. For each of these hemocytes, two regions of interest (ROIs) of approximately 0.5 um^2^ were plotted in the areas with the highest JC_agg_ fluorescence. For each plotted ROI, two values were calculated: the average JC_agg_ (red) fluorescence and the average JC_mon_ (green) fluorescence. The relative Δωm for a given ROI was calculated as the ratio JC_agg_ : JC_mon_. The Δωm for a given hemocyte was calculated as the average ratio for both ROIs. The values of a minimum of 10 hemocytes per hemolymph sample (20 hemocytes per scallop) were used for Δωm determination in a given animal. All image analyses were carried out using the software Fiji (Bethesda, MD, United States)^1^.

### Hemocyte Immune Activity

#### Determination of Circulating Hemocytes

The total number of circulating hemocytes was measured in the hemolymph of each experimental scallop. To do this, two samples of 10 μL each were mixed with 10 μL of PBS 1X and 10 μl of 0.4% Trypan-blue staining. In each sample the number of hemocytes was quantified using a Neubauer cell-counting chamber.

#### Determination of Infiltrating Hemocytes

A polyclonal antibody against scallop hemocytes was generated in CF-1 mice (4–6 weeks old). Hemocytes were obtained from scallops challenged with a sublethal dose (1 × 10^7^ CFU × mL^-1^) of the VPAP18 strain for 24 h (see section “Experimental Setup”). Then, total proteins from hemocytes were extracted and quantified as described below (see section “Hemocyte Protein Content”). For antibody production, mice were subcutaneously injected at 1, 15, and 30 days with 250 μg of an hemocyte protein extract diluted 1:1 in FIS peptide (peptide sequence: FISEAIIHVLHSR), a T helper cell activator (Prieto et al., 1995), and 1:1 Freund’s adjuvant (Thermo Scientific). The antiserum was collected on day 45, centrifuged at 700 *g* for 10 min and the supernatant was stored at -20°C. Antibody specificity was determined by western blot as described before (Schmitt et al., 2015) and the antibody capacity to detect hemocytes was determined in gills by immunofluorescence (Supplementary Figure 1).

Determination of the total number of infiltrating hemocytes in gonad tissues was performed by immunofluorescence analysis using the polyclonal antibody against whole hemocytes. For this, tissues were dissected under sterile conditions and fixed in Bouin’s solution (0.9% picric acid, 9% formaldehyde, 5% acetic acid). Fixed samples were dehydrated through an ascending ethanol series and embedded in Histosec (Merck). Sections were cut at 5 μm using a rotary microtome (Leica RM 2235) and mounted on glass slides. Paraffin sections were cleared in Neoclear (Merck) and hydrated by incubations in a descending ethanol series. Then, paraffin sections were incubated overnight at 4°C with the anti-hemocyte antibody (1:100), PBS with 1% BSA. A second incubation was performed for 1 h at 20°C with goat anti-mouse Alexa Fluor 568-conjugated (Thermo Scientific) 1:200 in PBS/1% BSA. Control slides were incubated with the prebleed serum of mouse. The slides where analyzed using a Leica TCS SP5 II spectral confocal microscope (Leica Microsystems) and images were obtained with a Leica 40 × 1.25 Oil HCX PL APO CS lens (Leica Microsystems), using the autofluorescence of the fixed tissue as contrast. The examination was performed on at least three tissue sections from different scallops to ensure they were consistently reproducible. The number of infiltrated hemocytes into gonad tissues was manually counted in 5 (400×) fields per scallop using the ImageJ v1.52e software.

#### Reactive Oxygen Species (ROS) Production

Reactive oxygen species production was measured in the hemolymph (where the number of hemocytes was previously quantified) of each experimental scallop using the nitroblue tetrazolium (NBT) reduction assay modified from Malham et al. (2003). To do this, two hemolymph samples of 100 μL each was obtained per scallop. These were incubated during 30 min in 96-well culture plates for hemocyte adhesion, time after which 15 μL of zymosan (Sigma; in 1% PBS, final concentration of 16 particles per hemocyte) was added and incubated for 15 min to allow hemocytes to phagocyte. The supernatant was then removed and 90 μL of PBS 1X plus 10 μL of NBT (1 mg/mL in 1% PBS) were added, and incubated for 90 min in darkness. Supernatant was removed and hemocytes fixed with 100% methanol 100 μL during 3 min, followed by one wash with 70% methanol 100 μL for 5 min. Then 120 μL of 2 M KOH was added to disrupt hemocyte membrane, plus 140 μL DMSO to solubilize the formazan produced by the NBT. Optical density (OD) at 630 nm was measured on a microplate spectrophotometer (EPOCH, BioTek) and the results expressed as OD values per million hemocytes^-1^. OD was corrected using wells that followed the same process but without hemocytes (negative controls).

#### Hemocyte Protein Content

Total protein was quantified in 0.03 g (wet mass) of hemocyte pellets from each scallop following Brokordt et al. (2015). Hemocytes were homogenized in 150 μL of homogenization buffer (32 mM Tris-HCl at pH 7.5, 2% SDS, 1 mM EDTA, 1 mM Pefabloc and 1 mM protease inhibitor cocktail; Sigma) and incubated for 5 min at 100°C. After this time, 100 μL of homogenization buffer were added to each sample and incubation was resumed for an additional 5 min. The homogenate was centrifuged at 10,600 *g* for 20 min. Total protein was quantified in a microplate spectrophotometer EPOCH (BioTek) at 562 nm in an aliquot of the supernatant using Micro-BCA kit.

### Immune Genes

Transcriptional levels of four immune-related genes were analyzed in the hemocytes from each experimental scallop. Two of these genes correspond to two antimicrobial effectors: *ferritin-1* (*Apfer1*; GenBank Accession No. KT895278) and *big-defensin* (*ApBD1*; GenBank Accession No. KU499992), which were already characterized and validated as immune genes for *A. purpuratus* by our research group (Coba de la Peña et al., 2016; Oyanedel et al., 2016b; González et al., 2017). In addition, two cDNA sequences showing homologies with immune sensors, such as a toll-like receptor (*ApTLR*; GenBank Accession No. MH732641) and a C-type lectin (*ApCLec*; GenBank Accession No. MH732642) were identified by next generation sequencing from *A. purpuratus* RNA by the Illumina HiSeq4000 platform (unpublished data) and included in this analysis.

#### RNA Extraction and First Strand cDNA Synthesis

RNA from hemocyte pellets was extracted using TRIzol^®^ reagent according to the manufacturer’s instructions (Thermo Scientific). RNA was then treated with DNAse I (Thermo Scientific) during 15 min at room temperature and inactivated by heat, 10 min at 65°C, followed by a second precipitation with sodium acetate 0.3 M (pH 5.2) and isopropanol (1:1 v:v). The RNA obtained was quantified using an Epoch spectrophotometer (BioTek, Winooski, VT, United States), and integrity was verified by the visual inspection of rRNA bands in electrophoretically separated total RNA. The RNA was stored at -80°C for further use.

Reverse transcription (RT) of RNAs from hemocytes was carried out by AffinityScript QPCR cDNA Synthesis Kit (Stratagene, Santa Clara, CA, United States) following the manufacturer’s protocol. RT of RNAs was done in equiproportions (i.e., from equal quantities of RNA) within all compared samples from each experiment.

#### Quantitative Real-Time PCR

The primers used in this study for quantitative real-time PCR (RT-qPCR) are listed in Table 1. Primers for the new *TLR* and *CLec* were designed using Primer3web version 4.1.0 (Untergasser et al., 2012) to have melting temperatures of 58 to 60°C and generate PCR products of 50 to 150 bp. For the *TLR*, the amplified region included a partial 3′-UTR sequence to ensure specific amplification of one *TLR* putative homolog. β-actin was used as endogenous control in order to normalize experimental results (Coba de la Peña et al., 2016; González et al., 2017).

**Table 1:** Nucleotide sequences of the primers used for RT-qPCR of immune and housekeeping genes in Argopecten purpuratus.

Gene	Sequences (5′–3′)	Amplicon (bp)	Reference
*ApTLR*	F: CGACAAAACAGAGAAACAAATGGC	95	Present study
	R: GTGAACCTCAGTCCGTCAATCT		
*ApCLec*	F: CCTATGAACTATGCCTGCCGAT	76	Present study
	R: TTGTCCATCCGTTACAACCCAT		
*Apfer1*	F: CATCACCAACCTGAAACGTGTT	69	Coba de la Peña et al., 2016
	R: TACTCCAGGGATTCTTTGTCGTACA		
*ApBD1*	F: TGCCGTGTTCCAGATGA	101	González et al., 2017
	R: TACTCCAGGGATTCTTTGTCGTACA		
*Ap β-actin*	F: GAATCTGGCCCATCCATTGT	65	Coba de la Peña et al., 2016; González et al., 2017
	R: CGTTCTCGTGGATTTTTTTCAAGT		

Each RT-qPCR reaction contained 10 μL of Takyon Low ROX SYBR 2X (Nalgene^®^), 2 μL cDNA and 0.3 μM (final concentration) of each primer, in a final volume of 20 μL. RT-qPCRs were run in a Real-Time PCR System Agilent Technologies (Stratagene MX3000P). Initial denaturation time was 3 min at 95°C, followed by 40 PCR cycles of 95°C, 15 s and 60°C, 30 s. After the PCR cycles, the purity of the PCR product was checked by the analysis of its melting curve; the thermal profile for melting curve analysis consisted of denaturation for 15 s at 95°C, lowered to 55°C for 15 s and then increased to 95°C for 15 s with continuous fluorescence readings. During RT-qPCR, the efficiency of gene amplification were approximately equal to that of the housekeeping gene (as it was determined by slope calculation) and the comparative C_T_ method (also called ΔΔC_T_ method) (Pfaffl, 2001) was applied for relative quantification. Experiments included eight biological replicates and three technical replicates were performed.

### Energetic Status

#### Muscle Carbohydrates

The energetic status of scallops at the three reproductive stages was evaluated through their content of total carbohydrates in the adductor muscle, the main energy storage tissue for these organisms (Brokordt and Guderley, 2004). To quantify total carbohydrates, samples of adductor muscles from each scallop were dried to a constant mass at 60°C. Dry tissues were then pulverized in a mortar and homogenized with deionized water at a proportion 1:1 (w/v). The phenol-sulfuric acid method described by Dubois et al. (1956) was used for total carbohydrate determinations. Its concentration was determined in a spectrophotometer (Variant Cary UV) at 490 nm using as standard a solution of glycogen in deionized water at a concentration of 50 μg mL^-1^.

### Statistical Analyses

Results are presented as means ± standard errors of the mean (SE). The Kolmogorov–Smirnov test was used to test normality while homoscedasticity was tested with the Levene test. Results meeting the requirements for parametric analyses were evaluated by a factorial analysis of variance (ANOVA), with the reproductive stage and challenge as factors, followed by an FDR-BY (false discovery rate) *post hoc* test (Benjamini and Yekutieli, 2001). When assumptions for parametric analyses were not met, a Kruskal–Wallis test was applied followed by *U*-Mann–Whitney pairwise comparisons. All statistical analyzes were conducted using SPSS 15.0 (SPSS, Inc., Chicago, IL, United States) and R version 3.3.1 (R Core Team, 2016) with the “agricolae” package (De Mendiburu, 2017).

## Results

### Hemocyte Metabolism

#### Hemocyte Respiration Rates

**FIGURE 1:**
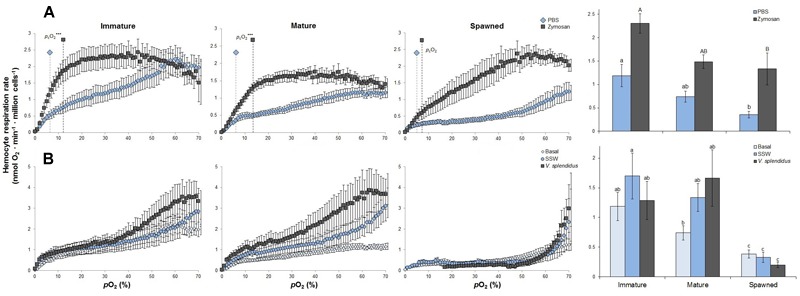
Effects of **(A)** *in vitro* challenge with zymosan and **(B)** *in vivo* challenge with *Vibrio splendidus* on hemocyte respiration rates and critical O_2_ (*p*_c_O_2_) (only for *in vitro*) from immature, mature, and spawned *Argopecten purpuratus* (*n* = 4–8 per condition and treatment). For bar graphs, values associated with the same letter belong to the same subset based on *a posteriori* multiple comparison test Student-Newman-Keuls. ^∗∗∗^*P <* 0.001.

*Ex vivo* experimentations showed that, compared to spawned scallops, hemocyte RR was between 2- and 3.5-fold higher in immature and mature animals, respectively (*F* = 6.135; *P* < 0.001). Compared to their control (PBS) values, at the 30–40% *p*O_2_ the zymosan challenge significantly increased hemocyte RR for immature (*F* = 11.983; *P* = 0.005), mature (*F* = 15.865; *P* = 0.001) and spawned scallops (*K* = 7.410; *P* = 0.006) by 1.9-, 2-, and 3.7-fold, respectively (Figure 1A). Post-challenge hemocytes from immature scallops showed the highest RR, and those from spawned the lowest RR (Figure 1A). The difference in RR between PBS and zymosan challenge increased with decreasing *p*O_2_. Critical *p*O_2_ values did not differ among hemocytes from different reproductive stages (*F* = 0.902; *P* = 0.424), and remained between 4 and 8% air saturation. However, challenging these hemocytes with zymosan caused *p*_c_O_2_ values to increase by roughly 2- and 3-fold in immature and mature scallops, respectively (*F* = 5.915; *P* = 0.011) (Figure 1A).

*In vivo* challenges evidenced that RRs varied among reproductive stages following the order immature = mature > spawned for all three treatments (basal: *K* = 6.400; *P* = 0.008; SSW: *F* = 4.830; *P* = 0.034; *V. splendidus: K* = 8.882; *P* = 0.012) (Figure 1B). In all cases, spawned scallops presented hemocytes with the lowest RRs, and *V. splendidus* challenged hemocytes in immature and mature animals had over sevenfold higher hemocyte RR than in spawned scallops. Treatment had no impact on RR of immature (*F* = 0.566; *P* = 0.579) mature (*K* = 3.714; *P =* 0.156) or spawned scallops (*F* = 1.732; *P* = 0.222) (Figure 1B).

#### Citrate Synthase Activity

**FIGURE 2:**
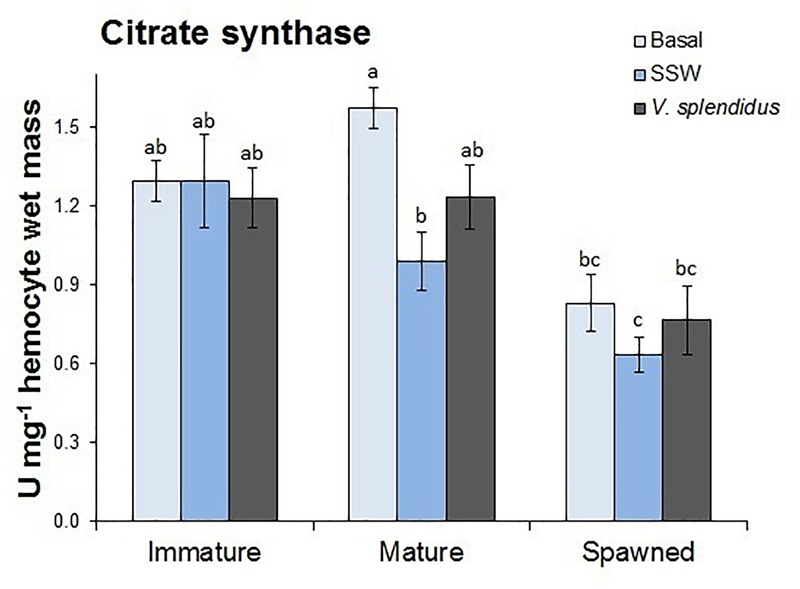
Effects of *in vivo* challenge with *V. splendidus* on citrate synthase (CS) activity in hemocytes form immature, mature, and spawned *A. purpuratus*. Values associated with the same letter belong to the same subset based on a FDR-BY (false discovery rate) *post hoc* test (Benjamini and Yekutieli, 2001) (*n* = 4–8 per condition and treatment).

The activity of the mitochondrial enzyme CS was measured in hemocytes and turned out to be different among scallops in the assessed reproductive stages (*F* = 17.32; *P* < 0.0001); but not between scallops at basal or injected either with *V. splendidus* or SSW (control) (*F* = 2.22; *P* = 0.118) (Figure 2). However, the interaction between the reproductive stage and immune status significantly affected hemocyte CS activity (*F* = 7.16; *P* < 0.0001). Regardless the immune condition, hemocytes from spawned scallops showed the lowest CS activity, while those from immature and mature scallops were similar. Under basal conditions, hemocytes from mature scallops showed the highest CS activity, but values were only significantly different from those obtained for SSW-injected mature scallops and spawned scallops under all immune conditions.

#### Hemocyte Mitochondrial Membrane Potential (Δωm)

**FIGURE 3:**
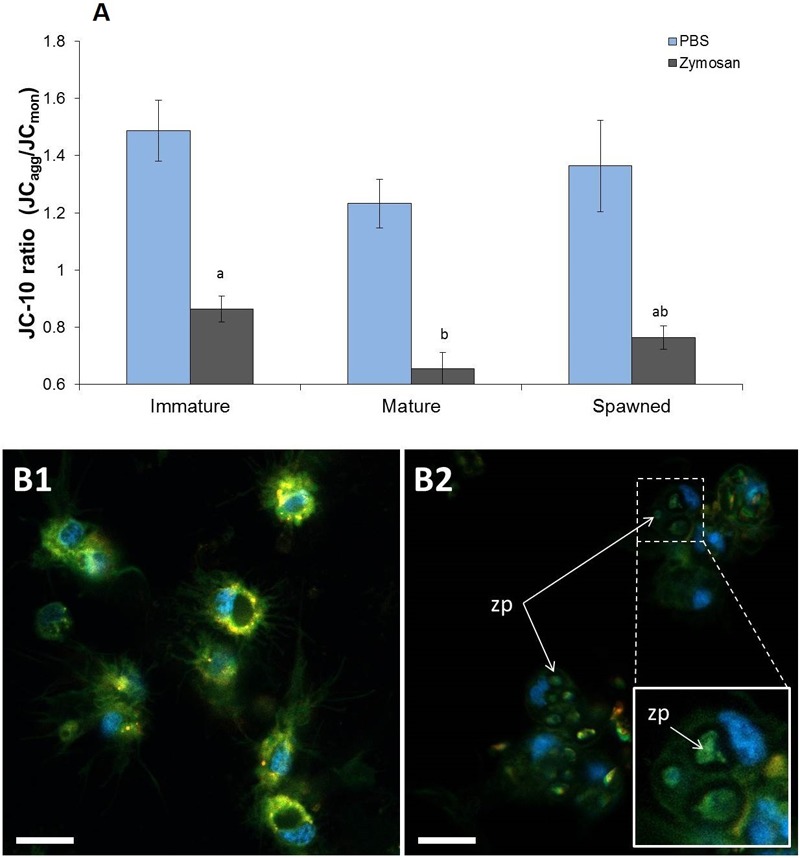
Mitochondrial membrane potential from *A. purpuratus* hemocyte obtained through JC-10 staining (red and green fluorescence). **(A)** Values associated with the same letter belong to the same subset based on *a posteriori* multiple comparison test Student-Newman-Keuls. Challenge with zymosan caused in all cases a decrease in JC-10 ratios. **(B)** Subpanels 1 and 2 show representative images of hemocytes respectively without and with zymosan challenge. Blue fluorescence corresponds to Hoechst 33342 staining (nuclei-specific). Scale bars = 10 μm. Zp = phagocyted zymosan particles (*n* = 4–8 per condition and treatment).

JC-10 ratio results evidenced that undisturbed hemocyte Δωm did not differ among animals in different reproductive stages (*F* = 1.233; *P* = 0.313), showing an overall JC-10 ratio average of 1.36 (Figure 3A). However, when these hemocytes were challenged with zymosan, JC-10 ratio values significantly decreased in all cases by an average of 40% (Figure 3A,B). Under challenged conditions, the hemocytes from mature scallops had significantly the lowest Δωm (*F* = 4.764; *P* = 0.021) (Figure 3A). As shown in Figure 3B, the confocal analyses also allowed verifying that hemocytes had successfully phagocyted zymosan particles. Although phagocytic rate was not quantified, observations indicate that most frequently hemocytes had 1–2 phagocyted particles and a maximum of 4.

### Hemocyte Immune Activity

#### Circulating Hemocytes

**FIGURE 4:**
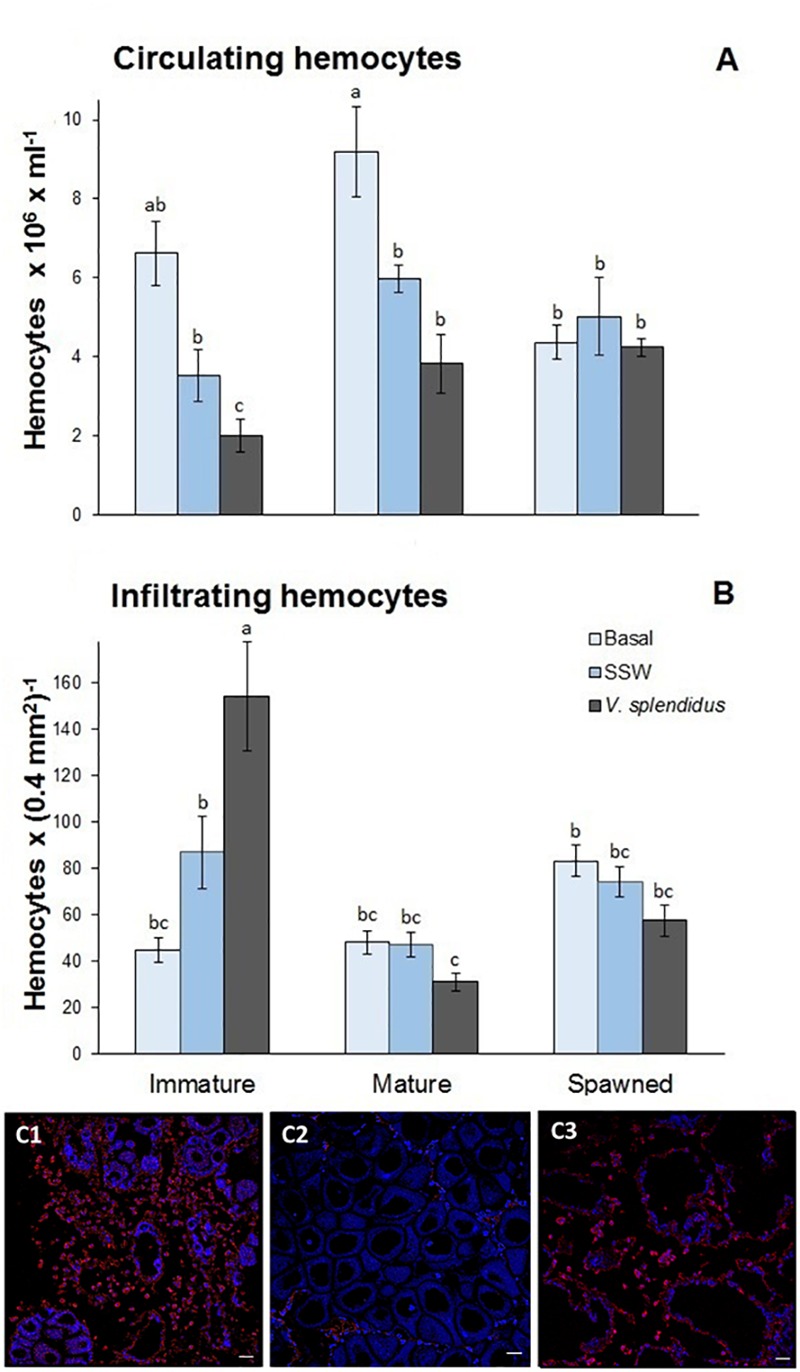
Effects of *in vivo* challenge with *V. splendidus* on **(A)** the density of circulating hemocytes and **(B)** the number of the infiltrating hemocytes in immature, mature, and spawned *A. purpuratus*. Values associated with the same letter belong to the same subset based on a FDR-BY (false discovery rate) *post hoc* test (Benjamini and Yekutieli, 2001). **(C)** Representative images for the detection of infiltrating hemocytes in gonad tissue from *V. splendidus*-challenged scallops at different reproductive stages by immunofluorescence and confocal analysis. Alexa 568 goat anti-mouse antibody was used for hemocyte detection (in red) and autofluorescence was used as contrast (in blue). **(C1)** Immature; **(C2)** mature; **(C3)** spawned. Magnification 400X, scale bar: 25 μm (*n* = 4–8 per condition and treatment).

Total circulating hemocytes (TCH) changed with the reproductive status, the immune treatment, and with the interaction of these factors (Figure 4A). In general, TCH was lower in spawned than in immature and mature scallops (*F* = 3.78; *P* = 0.048). Also, the lowest TCH was observed in scallops injected with *V. splendidus*, followed by SSW-injected animals at an intermediate level and finally, those in the basal status showing the highest amount of TCH (*F* = 14.09; *P* = 0.00002). Interestingly, only immature scallops decreased significantly TCH after *V. splendidus* injection (*F* = 2.60; *P* = 0.048), while mature and spawned scallops showed similar TCH to their injection control (SSW).

#### Infiltrating Hemocytes

Both the reproductive stage and its interaction with the immune treatment significantly affected the number of infiltrating hemocytes (respectively, *F* = 20.29, *P* < 0.0001; and *F* = 12.25, *P* < 0.0001) (Figure 4B). In general, the number of infiltrated hemocytes was the highest in immature scallops; among these, those scallops injected with *V. splendidus* showed the highest numbers (Figure 4B,C). Mature and spawned scallops showed lower amounts of infiltrating hemocytes with no apparent differences among scallops at basal status or injected with SSW or the bacteria.

#### ROS Formation

**FIGURE 5:**
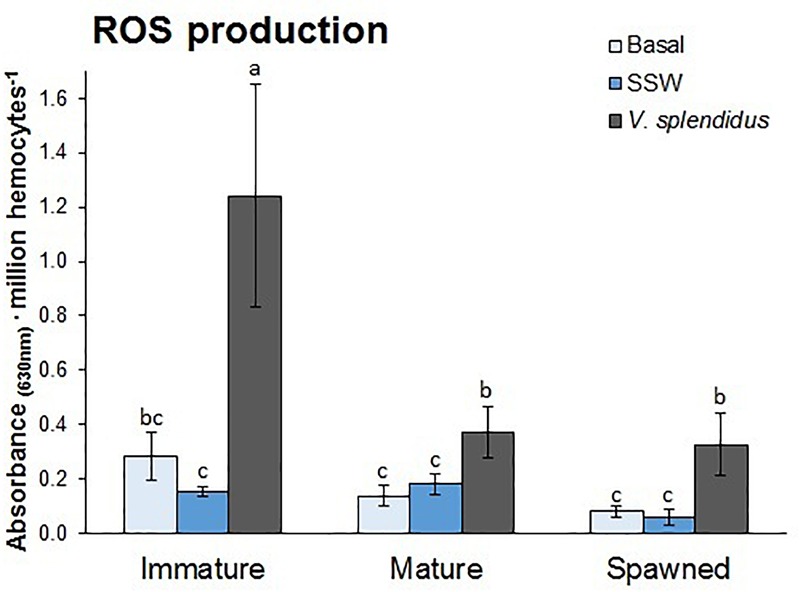
Effects of *in vivo* challenge with *V. splendidus* on reactive oxygen species (ROS) formation in hemocytes from immature, mature, and spawned *A. purpuratus*. Values associated with the same letter belong to the same subset based on a FDR-BY (false discovery rate) *post hoc* test (Benjamini and Yekutieli, 2001) (*n* = 4–8 per condition and treatment).

Reactive oxygen species production by circulating hemocytes was affected by the reproductive status and immune treatment, and the interaction between these factors (Figure 5). Independent of the immune treatment, ROS production was the highest in the hemocytes from immature, followed by mature and spawned scallops (*F* = 5.76; *P* = 0.006). Independent of the reproductive status, ROS production was higher in hemocytes from scallops injected with *V. splendidus*, and no difference was detected between hemocytes from undisturbed or injected with SSW scallops (*F* = 8.22; *P* = 0.0009). Interestingly, hemocytes from immature scallops showed threefold higher ROS production capacity than those from mature and spawned scallops after the bacterial challenge (*F* = 2.59; *P* = 0.047).

#### Hemocyte Protein Content

**FIGURE 6:**
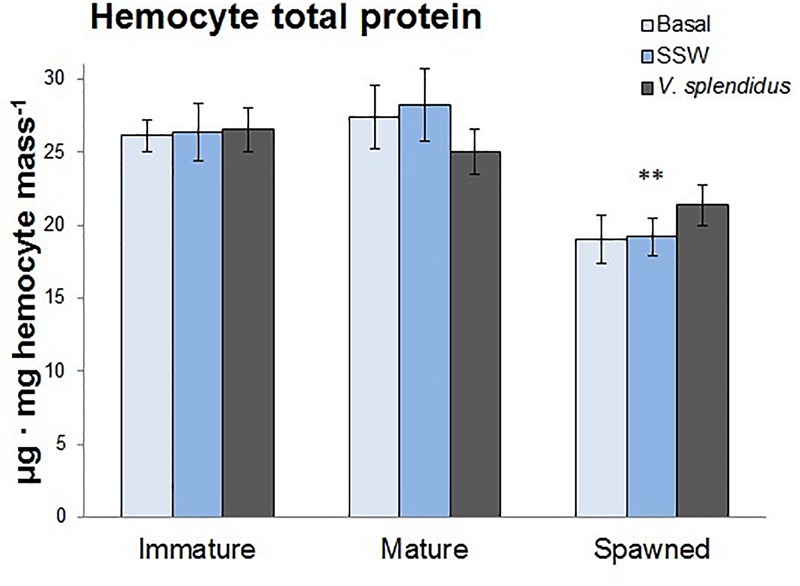
Effects of *in vivo* challenge with *V. splendidus* on hemocyte total protein content in immature, mature, and spawned *A. purpuratus* (*n* = 4–8 per condition and treatment; ^∗∗^*P* < 0.001).

The total protein concentration was assessed in circulating hemocytes as a proxy of their general energetic status. Only reproductive status significantly affected the protein content; being the hemocytes from spawned those with the lowest levels (*F* = 22.47; *P* < 0.0001) (Figure 6).

### Immune Genes

Transcriptional levels of the four immune genes evaluated in hemocytes varied significantly with the reproductive status, the immune treatments or with the interaction between them (Table 2).

**Table 2:** Two-way ANOVAs to compare transcriptional levels of immune genes between *Argopecten purpuratus* scallop at different reproductive status and immune treatments.

Source	DF	*F*	*P*	Comparisons
***ApTLR***				
Reproductive status (RS)	2	4.36	0.0192*	I > M = S
Immune treatment (IT)	2	1.91	0.1611	
RS × IT	4	2.75	0.0411*	
*ApCLec*				
Reproductive status (RS)	2	1.82	0.175	
Immune treatment (IT)	2	3.89	0.028*	V > SSW = B
RS × IT	4	3.35	0.017*	
***Apfer1***				
Reproductive status (RS)	2	7.00	0.00225*	I = M > S
Immune treatment (IT)	2	13.62	0.00002*	V > SSW = B
RS × IT	4	2.90	0.03212*	
***ApBD1***				
Reproductive status (RS)	2	3.69	0.0349*	I = M > S
Immune treatment (IT)	2	5.215	0.0103*	V > SSW = B
RS × IT	4	11.144	0.000006*	

*Reproductive status factor: immature (I), mature (M), and spawned (S) scallop reproductive stages; immune treatments: injected with *Vibrio splendidus* (V), injected with sterilized seawater (SSW), not injected-basal status (B). *n* = 4–8 biological replicates per condition (each replicate include three technical replicates). ^∗^*P* < 0.05.*

**FIGURE 7:**
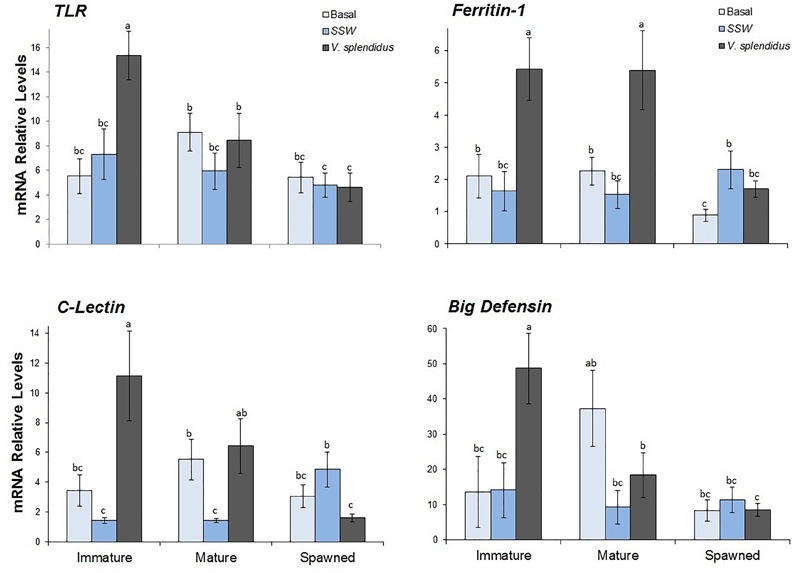
Effects of *in vivo* challenge with *V. splendidus* on transcriptional levels of immune-related genes in hemocytes from immature, mature, and spawned *A. purpuratus*. Values associated with the same letter belong to the same subset based on a FDR-BY (false discovery rate) *post hoc* test (Benjamini and Yekutieli, 2001) (*n* = 4–8 per condition and treatment).

Transcriptional levels of *ApTLR* were significantly affected by the reproductive status, and the interaction between this and the immune treatment (Figure 7 and Table 2). Regardless the immune treatment, immature scallops showed the highest *ApTLR* levels, the spawned individuals the lowest levels, and mature scallops was not different from either of these reproductive stages. The interaction analysis showed that only in immature scallop injected with *V. splendidus* transcriptional level of this immune receptor was significantly induced; while in mature and spawned scallop no differences of this gene was observed among immune treatments.

Relative levels of the *ApCLec* transcripts were affected by the immune treatment and the interaction between this and the reproductive status (Figure 7 and Table 2). Independent of the reproductive status, *ApCLec* transcriptional levels were higher in scallops injected with *V. splendidus* than in those injected with SSW; but the basal levels of this gene was similar to both injected treatments (*F* = 3.89; *P* = 0.028). The interaction analysis indicated that the levels of *ApCLec* was higher in immature *V. spendidus*-injected scallops. The injection with the bacteria also significantly induced the transcriptional levels of this gene in mature scallops, but only in comparison with their control SSW-injected. In contrast, in spawned scallops those injected with the bacteria showed the lowest levels of *ApCLec* transcripts, compared with the basal status or SWW-injected scallops.

The transcriptional levels of both effectors, *Apfer1* and *ApBD-1*, varied significantly with the reproductive stage, the immune treatment and the interaction between them (Figure 7 and Table 2). For both genes immature scallops showed the highest relative levels, while no difference was observed between mature and spawned scallops. Also for both genes, regardless the reproductive status, those scallops injected with *V. splendidus* showed higher transcript levels than those injected with SSW or not subjected to injection, and these did not differ between them. The interaction analysis indicated that the levels of *Apfer1* was highest in bacteria-injected immature and mature scallops. *ApBD-1* also showed the highest levels in immature scallops injected with the bacteria; but interestingly, after gonad maturation basal transcriptional levels of *ApBD-1* also increased greatly, largely overpassing the levels of immature scallops under basal status.

### Energetic Status

**FIGURE 8:**
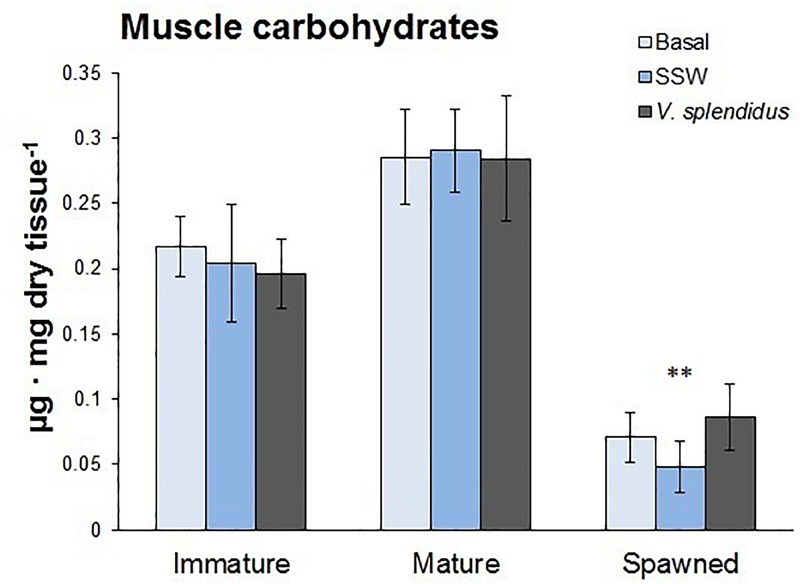
Total carbohydrate content in the adductor muscle of immature, mature, and spawned *A. purpuratus* (*n* = 4–8 per condition and treatment; ^∗∗^*P* < 0.001).

The energetic status of scallops at the three reproductive stages was evaluated through their carbohydrate reserves in the adductor muscle. Results showed that carbohydrate contents varied with the reproductive status (*F* = 53.9; *P* < 0.0001), but not with the immune status or the interaction between them (respectively, *F* = 0.47; *P* = 0.62; *F* = 0.59; *P* = 0.47) (Figure 8). Spawned scallops showed the lowest levels of carbohydrates in their adductor muscles, and no differences were observed between immature and mature scallops.

## Discussion

Empirical observations of reproduction-immunity tradeoffs have been made in some bivalve mollusks, mainly oysters (e.g., De Decker et al., 2011; Wendling and Wegner, 2013). However the hemocyte metabolic bases underlying this trade-off were not explored until now. As previously shown for oysters, in the present study we found a strong decrease in immune parameters after spawning in the scallop *A. purpuratus*, but the novelty of our results is that we additionally found that this decrease in their immune capacities was associated with a decrease in the aerobic metabolic capacity of hemocytes to mount immune responses at cellular and molecular levels. In general, our results showed that hemocytes from spawned scallops had lower respiration rates and capacity to produce ATP via the mitochondrial enzyme CS than hemocytes from immature and mature scallops at basal and after the bacterial challenge. This decreased metabolic capacity was paralleled with lower post-challenge hemocyte infiltration capacity and ROS production, as well as lower induction capacity of immune genes.

### Hemocyte Basal Energy Status Decrease After Spawning Impacting the Potentiality of the Immune Response

Carbohydrate reserves play a central role in supplying energy for gametogenesis, thus other studies conducted on scallops have shown a strong reduction in these energy reserves after both gonadal maturation and spawning (Brokordt et al., 2000a,b; Martinez et al., 2000; Brokordt and Guderley, 2004). In the present study we only observed a reduction in muscle carbohydrates after spawning. This is in agreement with what has been previously reported in several other bivalve species in which glycogen storages reach their minimain post-spawning individuals (e.g., Li et al., 2009; Hong et al., 2014). In some other cases, this was accompanied with a reduction in tissue adenylate energy charge (indicator of metabolic potential available to the cell) (Li et al., 2007). Overall, our results demonstrate that spawning is an energy demanding process that occurs at the expense of other tissue reserves and metabolic potential. Furthermore, we here revealed that low energy reserves also corresponded with post-spawning scallops (under undisturbed (basal) conditions) having hemocytes with significantly lower protein content and decreased metabolic rates, the later evidenced by low RRs (between 4.4- and 5.8-fold lower than mature and immature scallops), and CS activity (decreased by 0.8-times).

While the number of infiltrating hemocytes in the gonads did not differ from those in immature and mature scallops, post-spawning caused a reduction in the number of circulating hemocytes. The same was observed in the clam *Ruditapes philippinarum*, which was due to an increase in hemocyte mortality rates and an increase in their infiltration in the gonadal tissue (e.g., Li et al., 2009; Hong et al., 2014). This increase in basal levels of infiltrating hemocytes after spawning can be associated with the fact that hemocytes play also a role in gonad restructuration and gamete resorption (Cho and Jeong, 2005; Li et al., 2007; Samain et al., 2007; Wendling and Wegner, 2013). In our study the decrease in circulating hemocytes after spawning was not paralleled with an increase in infiltrating hemocyte abundance in the gonad, which may be associated with an increase in hemocyte mortality or a post-spawning decline in the hematopoiesis capacity. To this, we may add the reduction in the nutritional quality of circulating hemocytes in post-spawning *A. purpuratus*, which may be the result of a decrease in the available energy stored for hemocyte production and/or metabolic capacity for protein synthesis.

Regarding transcriptional levels of immune-related genes under basal conditions, results revealed that both immune receptors (*ApTLR* and *ApCLec*) did not change with the reproductive status. However, a post-spawning decrease in the antimicrobial effectors (*Apfer1* and *ApBD1*) transcripts was observed. To the best of our knowledge changes in transcriptional levels of immune-related genes during the reproductive process have not been assessed before in other bivalve mollusks. Nonetheless, similar to our results, Li et al. (2007) registered a marked decrease in the hemolymph antimicrobial activity in post-spawning *Crassostrea gigas* measured under both basal and heat-shock conditions.

Besides innate immunity, ferritins in mollusks are involved in different functional roles. Among others, they play a crucial task in iron storage and homeostasis, regulating intracellular iron concentration, an essential micronutrient (Coba de la Peña et al., 2016). In *Pecten maximus* it was demonstrated that hemocytes work in the transport of iron mediated by ferritins especially during gamete proliferation (Beninger et al., 2003). Therefore, the observed reduction in the basal levels of *ferritin* in hemocytes from post-spawning scallops could be associated with a decrease in the need for this micronutrient. Still, we cannot discard a reduction in the capacity to synthetizes this molecule.

Interestingly, transcriptional levels of *ApBD1* were strongly overexpressed under basal conditions in mature scallops compared to immature and spawned scallops. Such high levels of this anti-microbial peptide (AMP) could be associated with a potential maternal transfer of immunity (i.e., passive immunity), which is considered to play an essential role in protecting the offspring against pathogens at early stages of life (Wang et al., 2015). Maternal transfers of several immune molecules have been observed in *C. farreri* (Yue et al., 2013; Wang et al., 2015). On the other hand, the low levels of *ApBD1* mRNA transcripts observed in hemocytes from undisturbed immature and spawned scallops, may be associated with the preponderant immune function of this molecule as AMP, thus its induction is expected to occur mostly under a microbial exposure.

Overall, the assessed immune parameters under basal status indicate a post-spawning decrease in the structural components of the immune system (number of hemocytes) and their potential capacity of performing immune functions as indicated by reduced ATP producing machinery as it will be further discussed below. All these factors may well be pre-determining a decreased bio-energetic capacity for combatting pathogen exposure.

### Hemocyte Bioenergetics and Immune Associated Responses Are Lower After Gonad Maturation and Spawning

Hemocyte key bioenergetic parameters such as their oxygen consumption, CS activity (a key enzyme for the regulation of ATP production), as well as the potential protonmotive force driving ATP synthesis (i.e., Δωm), were here evaluated to assess how these may be constrained by reproduction and its energy-demanding processes.

In general, hemocytes from immature scallops showed the best metabolic and immune performances, while these severely decreased in undisturbed post-spawning scallops and in bacteria-challenged mature animals. A bioenergetic characterization of hemocyte response to a standardized challenge (here zymosan particles) allowed us to determine the potentiality of these cells to counteract pathogen exposure. Despite the reproduction stage, this challenge caused hemocytes to significantly increase their RRs, likely aimed at increasing ATP formation to respond to the increased energy requirements and which would be responsible for the ∼1.8-fold drop in Δωm. However, the respiratory behavior of hemocytes from immature and mature scallops was very different from the one of spawned scallops. The first two showed high (and stable) O_2_ consumption independent of environmental *p*O_2_ until around 20% *p*O_2._ Nevertheless, hemocytes from immature scallops maintained higher values than those from mature scallops, with average values of 2.3 to and 1.5 nmolO_2_ min^-1^ million cells^-1^, respectively. This response would allow hemocytes from these reproductive stages to maintain a sustained metabolic support for immune responses, in comparison with hemocytes from spawned scallops, which only maintain such levels of O_2_ consumption to a *p*O_2_ of ∼50%. Interestingly, hemocytes from immature scallops showed this post-challenge oxyregulatory-like behavior at ∼1.5-fold higher rates that hemocytes from mature scallops, which together with the higher Δωm suggest a better bioenergetical status for supporting immune responses.

*In vivo* evaluation of hemocytes evidenced that regardless the challenge status, spawned scallops presented the cells with the lowest RR. Remarkably, *V. splendidus* challenged hemocytes from immature and mature scallops had over sevenfold higher RR than hemocyte from spawned animals. This general pattern coincides with CS activity, albeit only a tendency was observed on its activity in hemocytes after bacterial challenge. Additionally, as under basal conditions hemocytes from injected immature and mature scallops presented higher nutritious status, as indicated by their levels of total protein content. These results suggest that hemocytes from immature and mature scallops have a higher metabolic capacity to support immune response upon bacterial exposure. Compared to *ex vivo* RRs, these values are nevertheless significantly lower, and despite the different nature of both challenges, this is probably in part due to the fact that *in vivo* measures were done 24 h-post challenge where the energy-demand for immune responses are likely reduced. Thus, both *in vitro* and *in vivo* are here providing a good and complementary overview of the metabolic requirements of setting the immune response across time. To our knowledge, there are no other studies addressing hemocyte RR as a result of an *in vivo* challenge. However, contrarily to our results, a previous work by Cheng (1976) using hemocytes phagocytizing *ex vivo* heat-killed vegetative cells of *Bacillus megaterium* did not register any increase in hemocyte RR.

In their immune role during the infection process, hemocytes (and namely the number of circulating/infiltrating cells) provide a good overview of the degree of the immune response. As a result of an infection, a negative correlation between the density of circulating and tissue-infiltrating hemocytes after infection has been reported in several bivalves (Paillard et al., 1996; Cochennec-Laureau et al., 2003; Donaghy et al., 2009; Allam and Pales-Espinosa, 2016). This would be the result of the active movement of hemocytes toward the infection sites leading to an increase in tissue-infiltrated hemocytes; which would be generalized in the case of systemic infections such as those caused by most *Vibrio* species (Allam and Pales-Espinosa, 2016). In the present study the effects of exposure to *V. splendidus* on the number of circulating and infiltrating hemocytes was only evident in immature scallops, with the first decreasing by nearly twofold and the second increasing also in the same proportion. Compared with basal levels, *Vibrio*-challenged mature scallops also decreased the density of circulating hemocytes, but this was similar to the response to the injection with SSW (injury control). Curiously, this decrease in circulating hemocytes was not paralleled with an increase in infiltrating hemocytes as in immature scallops. This suggests that hemocytes in mature scallops have a lower infiltrating capacity in the gonad than immature scallops, although a higher infiltration in other tissue cannot be ruled out. Post-spawning scallops showed lower levels of both circulating and infiltrating hemocytes, with no apparent bacteria-induced changes, suggesting that hemocytes from scallops at this stage were less actively responding to the bacterial challenge, which would be coherent with their lower metabolic capacities.

The production of ROS by immune-stimulated hemocytes has been recognized in various bivalves (e.g., Pipe, 1992; Lambert et al., 2003; Buggé et al., 2007) as agents of internal defense, damaging potential pathogens during phagocytosis process. Our results in *A. purpuratus* showed that ROS production by hemocytes increased as a consequence of *V. splendidus* exposure in all reproductive stages, but the magnitude of this response was fairly different among them. After bacterial challenge, hemocytes of mature and spawned scallops increased their ROS production by 1- and 3-fold, respectively, whereas in comparison with the control conditions (SSW injection), it augmented by sevenfold in immature animals. Overall, both of the cellular immune parameters assessed reveal the hemocytes from immature scallops as having the best response.

Molecular immune responses were examined through the transcriptional levels of two hemocyte pattern recognition receptors (*ApTLR* and *ApCLec*) and two anti-microbial effectors (*Apfer1* and *ApBD1*). In congruence with cellular responses, results exhibited that hemocytes from immature individuals induced the highest values after the bacterial challenge, most often followed by mature animals and with spawned scallops presenting the lowest values. Both receptors tested (*ApTLR* and *ApCLec* where here identified in *A. purpuratus* for the first time) were overexpressed after the *Vibrio*-challenge, but only when animals where immature. These results suggest that these receptors would be an important recognition component, potentially involved in the anti-bacterial immune defense of *A. purpuratus*. Interestingly, under challenge conditions, the AMP *ApBD1* transcriptional pattern followed a similar pattern of that shown by *ApTLR*. We recently showed that the expression of this AMP is regulated via a putative Rel/NF-kB signaling pathway in *A. purpuratus* (Oyanedel et al., 2016b), and present results suggest that this could be further mediated through the *ApTLR*. TLR signaling pathways have been found to mediate expression of antimicrobial effectors in other bivalve mollusks including scallops (Wang et al., 2011). Even so, herein we would like to bring up the importance of considering the animal reproductive status when characterizing immune pathways.

Like TLRs, lectins are critical components of innate immunity because of their pathogen pattern recognition and binding functions (Vasta et al., 2004). In scallops, a C-type lectin has only been previously characterized in *C. farreri* (Yang et al., 2010, 2011). Remarkably, these authors found that in addition to its recognition role, CfLec-1 also enhances the opsonization of hemocytes for the clearance of bacteria (Yang et al., 2011). In the present study, hemocytes from immature and mature scallops showed significant transcript inductions of *ApCLec* (in mature status only respect to their injury control) after the *Vibrio*-challenge. Thus, the expression profiles of isolated lectin suggest a functional capacity of these receptors during infectious challenge, but at a lower level in hemocytes from mature and not in post-spawning scallops.

Among their multiple functions, ferritins are involved in microbial elimination through an iron-withholding strategy: by storing host iron and preventing iron acquisition, bacterial growth, and proliferation is thereby inhibited (Ong et al., 2006). Ferritin induction by bacterial challenge has also been observed in other scallops such as *Mizuhopecten yessoensis* (Zhang et al., 2013; Sun et al., 2014). In a previous study we isolated and characterized *Apfer1*, and further reported its induction in response to inactivated *V. splendidus* (Coba de la Peña et al., 2016). The results of this study now add to the previous by showing that the induction of this gene also occurs upon the challenge with live *V. splendidus*, but only in hemocytes from immature and mature scallops.

Overall results regarding the immune-genes suggest a reduced molecular immune capacity in scallops after gonadal maturation and spawning, which is consistent with a reduced energy metabolic capacity of hemocytes under these stages. Energetically speaking this is not surprising, since gonadal maturation and spawning are energy-hungry processes, and a trade-off between reproduction and immunity would decrease the energy available for immune-related tasks. However, for post-spawning scallops, were the decrease in the immune capacity is most evident, we cannot rule out other regulatory mechanisms potentially underlying this trade-off such as signaling pleiotropy. In this regards, we suspect that the physiological trade-off between spawning and immunity in scallops may be regulated by neuroendocrine signals. For example, the spawning process of *A. purpuratus* has been shown to be modulated by the neuroendocrine system and specifically by catecholaminergic regulation (Martínez et al., 1996). This study revealed a striking increase of the catecholamines dopamine (DO) and norepinephrine (NE) during and after gamete release. Also a catecholaminergic regulation of immune response has been recently showed for oysters and scallops (Zhou et al., 2011; Song et al., 2015; Wang et al., 2018). In oysters, NE induces negative effects on hemocyte phagocytosis by means of their receptors (reviewed by Wang et al., 2018). Furthermore, a complete immune-related pathway from receptors to effectors has been recently shown to be modulated by microRNAs interacting with the neuroendocrine immunomodulation system (Chen et al., 2015). In scallops, Zhou et al. (2011) showed that high concentration of NE and epinephrine repressed the increase of antioxidants (superoxide dismutase and catalase) and lysozyme activities in hemolymph after a *V. anguillarum* challenge. Moreover, it has been observed that the α-adrenergic receptor on the surface of scallop hemocytes could be regulated at the transcriptional level during the immune response (reviewed by Song et al., 2015). It could transmit signals to regulate hemocyte phagocytosis and antibacterial function through the second messenger cAMP, and further modulate the induction of immune-related genes and the activity of immune-related enzymes in hemolymph (reviewed by Song et al., 2015). This information suggests that an increase of DO and/or NE during spawning of *A. purpuratus* could have reduced the immune activity of hemocytes and the transcriptional levels of immune-related genes, therefore further research is necessary to give light into this potential source of immune modulation.

### Post-spawning Immune-Decrease: Role in Massive Mortality Events?

*Argopecten purpuratus* wild and cultured populations are currently experiencing large mortality events throughout its distribution range. These have been observed to often coincide with the spawning period of the species (see Brokordt et al., 2015 and references therein) but despite the economic importance of *A. purpuratus* cultures (PromPerú, 2014; FAO, 2016), the reasons behind these spawning-associated mortality events have not yet been elucidated.

The present study evidenced how reproduction is a period during which energy and immunity is significantly reduced, making mature and specially spawned *A. purpuratus* scallops more prone to pathogenic infection as it occurs in other bivalve species (Li et al., 2010; Hong et al., 2014). *Vibrio* is responsible for massive mortalities during the reproduction period in mollusks across the world (e.g., Travers et al., 2009) as well as being a primary pathogen to many marine organisms (Reichelt et al., 1976). Thus, our results using *V. splendidus* provide an excellent basis for studying the role of this immunity-reproduction trade-off in the mortality events and elaborating adequate conservation measures targeting the amelioration of the bioenergetic capacity of the animals.

## Ethics Statement

This study was carried out in accordance with the recommendations of the CCAC guidelines (https://www.ccac.ca/Documents/Standards/Policies/Ethics_of_animal_investigation.pdf). The protocol was approved by the CEAZA Bioethics Committee and supervised by CONICYT Bioethics Committee.

## Author Contributions

KB and PS contributed to the conception and design of the study. YD, IE, CC, GR-I, EdlF-O, and LM contributed to the acquisition of data. CC, YD, IE, and GR-I organized the database and performed the statistical analysis. KB and GR-I worked on analysis, interpretation of data, and wrote the first draft of the manuscript. PS wrote sections of the manuscript. All authors contributed to manuscript revision, read, and approved the submitted version.

## Conflict of Interest Statement

The authors declare that the research was conducted in the absence of any commercial or financial relationships that could be construed as a potential conflict of interest.

## Footnotes

1 https://fiji.sc/

## Supplementary Material

Figure S1 Validation of whole hemocytes antibody detection by Western blot and Immunofluorescence. **(A)**, Determination of antibody specificity against total protein extract from hemocytes 1. SDS PAGE, 2. Western blotting. **(B)**, merged confocal images for detection of infiltrating hemocytes in scallop gills by immunofluorescence. In blue, nuclei. In green, hemocyte detection. Scale bar, 7 μm.
