# Supplementary figures and images for: Reproduction Immunity Trade-Off in a Mollusk: Hemocyte Energy Metabolism Underlies Cellular and Molecular Immune Responses
Source: Front Physiol. 2019 Feb 11;10:77. doi: 10.3389/fphys.2019.00077 (PMC6378683; doi:10.3389/fphys.2019.00077)

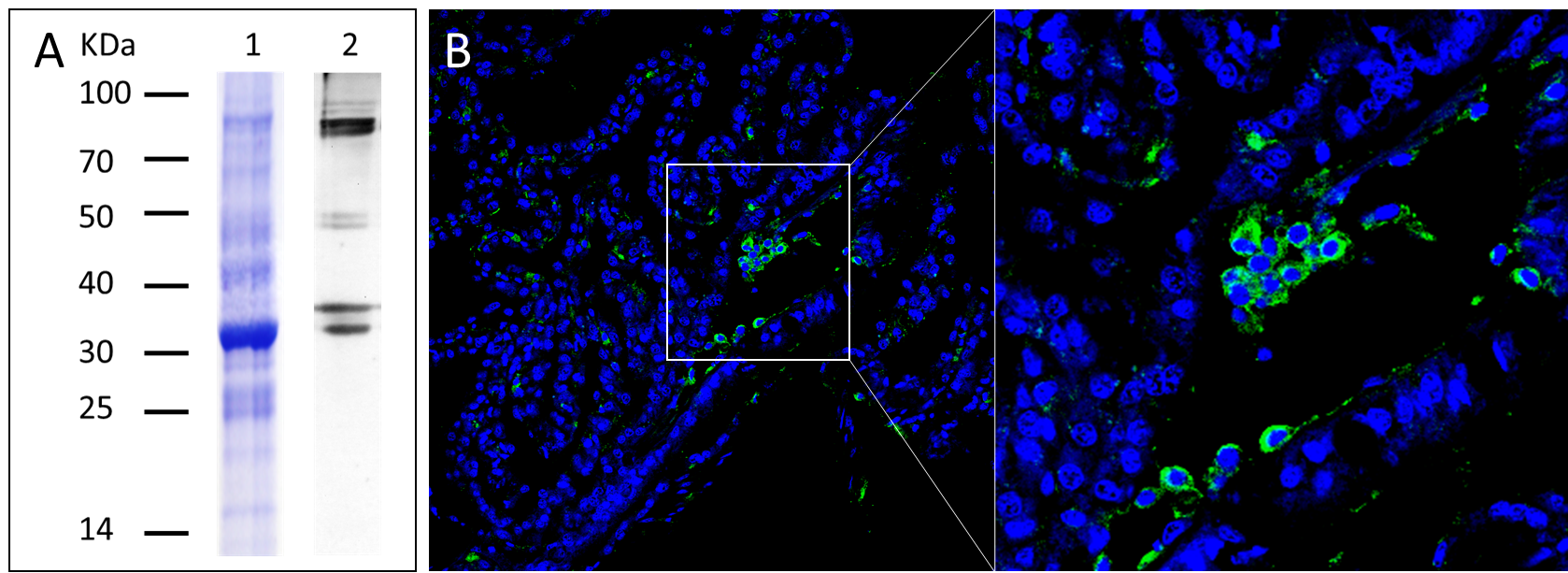

Supplement: Figure S1 — Validation of whole hemocytes antibody detection by Western blot and Immunofluorescence. (A), Determination of antibody specificity against total protein extract from hemocytes 1. SDS PAGE, 2. Western blotting. (B), merged confocal images for detection of infiltrating hemocytes in scallop gills by immunofluorescence. In blue, nuclei. In green, hemocyte detection. Scale bar, 7 μm. [file Image_1.png]
